# Neutrophil-Lymphocyte Ratio in Patients with Hypertriglyceridemic Pancreatitis Predicts Persistent Organ Failure

**DOI:** 10.1155/2022/8333794

**Published:** 2022-03-16

**Authors:** Zhihua Lu, Xiangping Chen, Huiqing Ge, Man Li, Binbin Feng, Donghai Wang, Feng Guo

**Affiliations:** ^1^Department of Critical Care Medicine, Sir Run Run Shaw Hospital, Zhejiang University School of Medicine, Hangzhou, Zhejiang, China; ^2^Department of Respiratory Care, Sir Run Run Shaw Hospital, Zhejiang University School of Medicine, Hangzhou, Zhejiang, China

## Abstract

**Background:**

The neutrophil–lymphocyte ratio (NLR) has been proposed as a surrogate marker of inflammation with prognostic value in various diseases. Our objective was to investigate the predictive value of the NLR as an indicator of persistent organ failure (POF) in patients with hypertriglyceridemic pancreatitis (HTGP).

**Methods:**

We retrospectively reviewed the data from patients with HTGP between 2016 and 2019. The NLR was obtained at admission. The diagnostic performance of the NLR for POF was evaluated by the area under the receiver operator characteristics curve (AUROC). Multivariate logistic regression determined whether elevated NLR was independently associated with POF.

**Results:**

Of the 446 patients enrolled, 89 (20.0%) developed POF. Patients with POF showed a significantly higher NLR than those without POF (*P* < 0.001). A positive trend for the association across increasing NLR quartiles and the incidence of POF was observed (*P*_trend_ < 0.001). The AUROC of NLR to predict POF was 0.673 (95% confidence interval, 0.627-0.716). With a cut-off of NLR > 6.56, the sensitivity and specificity were 73.0% and 55.7%, respectively. Multivariate analysis suggested that high NLR (>6.56) was independently associated with POF (odds ratio, 2.580; 95% confidence interval, 1.439-4.626; *P* = 0.001). Patients with a high NLR (>6.56) had a worse overall clinical course in HTGP.

**Conclusion:**

Elevated NLR was significantly associated with an increased risk of developing POF and could be an early independent predictor of POF in patients with HTGP.

## 1. Introduction

Hypertriglyceridemia is a well-established cause of acute pancreatitis (AP), which is the second etiological factor next to gallstones in China [1]. It is hypothesized that overproduction of free fatty acids from triglyceride metabolism leads to a cytotoxic effect on acinar and vascular endothelial cells, resulting in local and systemic inflammation [2]. Hypertriglyceridemic pancreatitis (HTGP) differs from other etiology as it has a more severe clinical course and less favorable outcomes [3]. Recent studies have identified that persistent organ failure (POF) (defined as organ failure lasting ≥48 hours) is the key determinant of the severity of AP regardless of the presence or absence of local pancreatic complications [4]. Therefore, early identification of patients who are likely to develop POF would help physicians select those patients who would benefit the most from close monitoring or aggressive treatments [2].

A variety of scoring systems has been identified to predict the severity and prognosis of AP, such as the Ranson's score, acute physiologic assessment and chronic health evaluation II (APACHE II) score, bedside index for severity in acute pancreatitis (BISAP) score, and sequential organ failure assessment (SOFA) score [5, 6]. There have been criticisms regarding their time-consuming and cumbersome to perform in routine clinical practice. It is also acknowledged that there is no one perfect scoring system to risk stratify and predict mortality in AP patients. Each scoring system has inherent strengths and weaknesses [7]. In several studies, simplified serum markers such as admission hematocrit, C-reactive protein (CRP), interleukin-6, interleukin-8, triglycerides, and urea nitrogen have been applied to predict the prognosis or severity of AP, but are expensive, not validated in the clinical field, and are not readily available [8–10].

The neutrophil-lymphocyte ratio (NLR) is inexpensive and easily derived from a routine complete blood count. NLR is dynamic and is influenced by inflammatory cytokines and the endocrine effects of the hypothalamic-pituitary axis [11]. Several studies have reported the value of NLR in predicting disease severity and outcomes in a variety of diseases, including systemic inflammatory diseases, cardiovascular diseases, and neoplastic states [12–14]. In AP specifically, some studies have shown that increased NLR is associated with severe AP and adverse outcomes [15]. However, there is limited data on the prognostic value of NLR in HTGP in the literature. This study is aimed at investigating the significance of NLR in predicting POF in patients with HTGP and further determining the optimal threshold for a high NLR in this population of patients.

## 2. Materials and Methods

### 2.1. Study Design and Patients

We performed a retrospective review of the electronic medical records of patients treated at the Sir Run Run Shaw Hospital, Zhejiang University School of Medicine, from January 2016 to December 2019. The Ethics Committee of Sir Run Run Shaw Hospital approved the study (protocol number: 20201026-31). Informed consent was waived as this was a retrospective review of the chart.

Patients were eligible for inclusion in the study if they had a clinical diagnosis of HTGP. The diagnosis of HTGP met all the following criteria: (a) AP diagnosis requires two or more of the following three criteria: acute onset of the characteristic AP pain; elevated serum lipase or amylase above three times the upper limit of normal; characteristic AP findings on imaging [16], (b) serum triglyceride level of ≥11.3 mmol/L at admission, or between 5.65 and 11.29 mmol/L accompanied by lactescent serum in the absence of other causes of pancreatitis [17]. Patients younger than 18 years, patients who presented in the hospital more than 72 hours after the onset of symptoms, patients who were transferred from other institutions, patients who had an etiology of AP in addition to hypertriglyceridemia, such as gallstones, chronic substantial alcohol use (≥4-5 drinks daily, over 5 years of ongoing), or binge drinking within a week before admission, and patients with missed data for analysis were excluded from the analysis.

### 2.2. Data Collection

We extracted patients' data from a review of patients' electronic medical records. Demographic data, main preexisting comorbidities, time from the onset of pain to admission, laboratory test results, organ failure, organ support therapy, length of hospital stay, and patient death during hospitalization were collected. Blood samples for hematological and biochemical data were obtained within one hour of presentation and analyzed within 6 hours in the same laboratory. CT results were reviewed to identify local pancreatic complications. The Ranson's score, the APACHE II score, the BISAP score, and the SOFA score were calculated. At the time of data collection, collectors were blinded to the outcomes being investigated.

The NLR was determined by calculating the ratio between absolute neutrophil and lymphocyte counts obtained from the same blood sample collected at the time of presentation of the patient to the hospital.

### 2.3. Outcomes and Definitions

The primary clinical endpoint was the occurrence of POF. Organ failure was defined as having a score of ≥2 in one or more of the three organ systems described in the modified Marshall score: respiratory failure if the ratio of PaO2/FiO2 was <300 mm Hg, renal failure if serum creatinine was ≥1.9 mg/dL, and cardiovascular failure if systolic blood pressure was <90 mm Hg not responsive to fluid resuscitation. POF was defined as organ failure that persisted for more than 48 hours. That definition was following the revised Atlanta definition criteria [16].

### 2.4. Statistical Analysis

Continuous variables were not normally distributed and presented as medians with interquartile range (IQR) and compared using the Mann–Whitney *U* test. Categorical variables were presented as frequencies and proportions (%) and compared using the *χ*^2^ test or Fisher's exact test, as appropriate. Linear-by-Linear Association test was performed to investigate the trend of the development of POF across increasing NLR quartiles. The area under the receiver operator characteristics curve (AUROC) was used to evaluate the discriminative ability of NLR for POF in HTGP. This was also used to assess the optimal cut-off, by showing the trade-off between sensitivity and specificity. Subsequently, a univariate analysis was performed to determine the association of the proposed clinical risk factors with POF. Variables that were statistically significant in the univariate analysis were selected for further multivariate logistic regression analysis to identify independent predictors of POF. Odds ratios (OR) and 95% confidence intervals (CI) were presented. A *P* value < 0.05 was considered statistically significant. Statistical analysis was performed with IBM SPSS Statistics for Windows, Version 22.0 (IBM Corp, Armonk, NY).

## 3. Results

### 3.1. Patient Characteristics and POF for Different Levels of NLR

**Figure 1:**
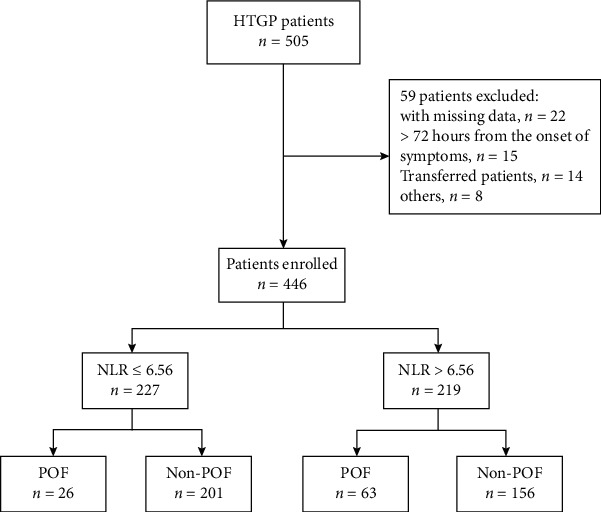
Flow chart of patient enrollment. HTGP: hypertriglyceridemia-induced acute pancreatitis; NLR: neutrophil-lymphocyte ratio; POF: persistent organ failure.

During the study period, 505 patients fulfilled the diagnostic criteria of HTGP. After exclusion, 446 patients were included in the study (Figure 1). There were a total of 89 patients (20.0%) who developed POF. The demographic and clinical characteristics of patients with and without POF were demonstrated in Table 1. The POF cohort had a long time from pain onset to admission compared to the non-POF group (*P* < 0.001). Patients with POF presented higher values of white blood cells (*P* = 0.006), neutrophil (*P* = 0.002), NLR (*P* < 0.001), hematocrit (*P* = 0.003), triglyceride (*P* = 0.005), urea (*P* = 0.012), glucose (*P* = 0.001), and CRP (*P* < 0.001) at admission, and lower levels of lymphocyte (*P* = 0.006), calcium (*P* < 0.001), and albumin (*P* < 0.001). Scoring systems such as the Ranson's score, APACHE II score, SOFA score, and BISAP score showed statistical differences between patients with and without POF (all *P* < 0.001).

**Figure 2:**
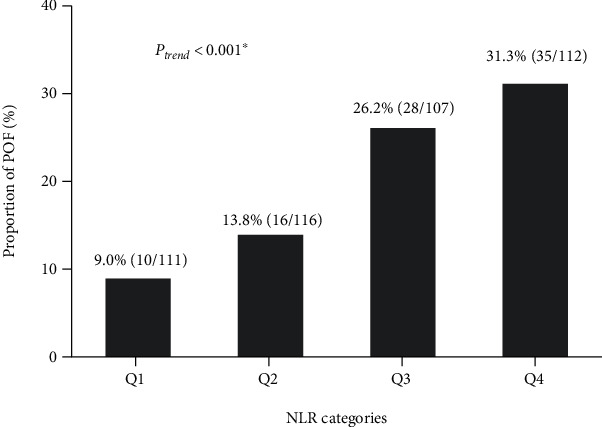
The proportion of POF for the quartiles of NLR at admission. Q1: NLR ≤ 4.386; Q2: 4.386 < NLR ≤ 6.563; Q3: 6.563 < NLR ≤ 10.333; Q4: NLR > 10.333. ∗The Linear-by-Linear Association test for the POF trend was significant. NLR: neutrophil-lymphocyte ratio; POF: persistent organ failure.

The median NLR at admission was 6.56 (IQR, 4.39-10.3). For comparison purposes, included patients were stratified into quartiles according to their admission NLR: quartile 1 (NLR ≤ 4.386), quartile 2 (4.386 < NLR ≤ 6.563), quartile 3 (6.563 < NLR ≤ 10.333), and quartile 4 (NLR > 10.333). As shown in Figure 2, POF developed in 9.0% (10/111) patients with the first quartile of NLR and 31.3% (35/112) with the highest quartile. There was a statistically significant trend for increasing the incidence of POF with a higher NLR level (*P*_trend_ < 0.001).

### 3.2. Admission NLR as an Early Predictor of POF

**Figure 3:**
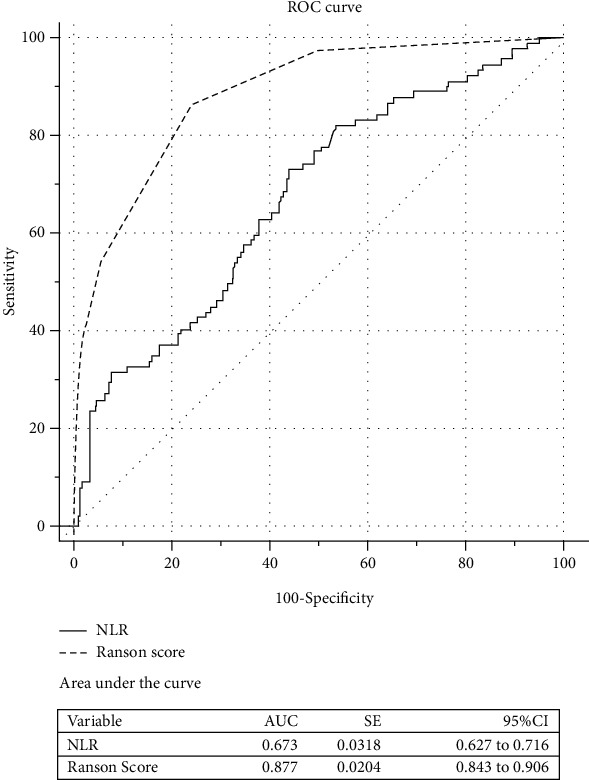
Area under receiver-operator curves of the NLR and Ranson's score to predict the development of persistent organ failure. AUC: area under the curve; CI: confidence interval; NLR: neutrophil-lymphocyte ratio; ROC: receiver-operator characteristic curves; SE: standard error.

We calculated the AUROCs of NLR and Ranson's score for predicting POF in HTGP patients (Figure 3). NLR had an AUROC of 0.673 (95% CI, 0.627-0.716; *P* < 0.001), and Ranson's score had an AUROC of 0.877 (95% CI, 0.843–0.906; *P* < 0.001). The optimal cut-off value of NLR was 6.56, corresponding to a sensitivity of 73.0%, a specificity of 55.7%, a positive likelihood ratio of 1.65, and a negative likelihood ratio of 0.48.

As shown in Table 2, the univariate analysis revealed that prior AP, the time from the onset of pain to admission, NLR, calcium, urea, triglycerides, white blood cells, hematocrit, albumin, CRP, and glucose were significantly associated with the development of POF. Then, these characteristics that showed statistical differences above were performed in multivariable analysis. The NLR level (>6.56) was independently associated with the development of POF (OR, 2.580; 95% CI, 1.439-4.626; *P* = 0.001).

### 3.3. Clinical Course by NLR Subcategorization

The patients were dichotomized into low NLR (≤6.56) and high NLR (>6.56) based on cut-off values. Patients with high NLR were more likely to progress to a severe state determined by the revised Atlanta classification compared to patients with low NLR (*P* < 0.001). Greater need for organ support (ventilation support, *P* = 0.003; renal replace treatment, *P* = 0.001), more ICU admission (*P* = 0.001), higher incidence of local complications (acute peripancreatic fluid collection/pancreatic pseudocyst, *P* < 0.001; acute necrotic collection/walled-off necrosis, *P* = 0.001), and longer hospital stays (*P* < 0.001) were also associated with high NLR. There were no differences in vasoactive agents, the incidence of infected pancreatic necrosis, need for percutaneous catheter drainage or surgical necrosectomy, or hospital mortality between the NLR groups (Table 3).

## 4. Discussion

This study demonstrated that HTGP that experienced POF presented higher values of NLR at admission. There was a positive trend for the association between increasing NLR categories and the incidence of POF. The AUROC for prognosticating POF in HTGP was remarkable for the NLR. After dichotomization based on an optimal cut-off point of 6.56, multivariate analysis showed that the higher level of NLR was an independent predictive factor for POF in HTGP. Furthermore, elevated NLR was associated with an adverse clinical course of HTGP. Therefore, the results of our study suggested that NLR could be an early indicator to predict POF in HTGP and was able to effectively differentiate patients who experience mild and severe AP.

NLR represents a balance between inflammatory activator neutrophils and inflammatory regulator lymphocytes. Neutrophils propagate and promote inflammation through activation of a cascade of inflammatory cytokines (interleukin-1, interleukin-6, and TNF-*α*) and proteolytic enzymes (myeloperoxidase, elastase, and collagenase). These inflammatory mediators have been shown to play a significant role in the development of systemic inflammatory response syndrome (SIRS) and the progression to multiple organ dysfunction syndromes (MODS) [18]. On the contrary, lymphocytes are related to the regulation of the immune system pathway, which regulates subsequent systemic inflammation as the disease progresses. In the setting of uncontrolled inflammation, lymphopenia occurs as a result of lymphocyte apoptosis and redistribution [19]. Persistent lymphopenia is an independent marker of progressive inflammation in emergency admission and intensive care patients [20]. Previous studies have shown that elevated NLR is a reliable and sensitive inflammatory marker that can be a valuable predictor of poor outcomes in benign and malignant conditions [21].

AP is an inflammatory process in which local pancreatic injury leads to systemic inflammation through the activation of cytokine cascades [22]. A growing number of studies have shown that NLR is associated with AP and is better than other serum markers in predicting the severity and prognosis of AP. Azab et al. were the first to demonstrate that NLR was superior to total leucocyte count in predicting ICU admission or prolonged hospitalization for AP [23]. Suppiah et al. revealed that NLR was significantly associated with the risk of developing a severe form of AP [24]. To date, there have been very few studies on the clinical significance of NLR in patients with HTGP. Wang et al. retrospectively reviewed a cohort of 110 patients with HTGP and concluded that NLR measured in the first 48 hours had the highest discriminatory capacity for severe HTGP among the inflammatory biomarkers [25]. In line with their study, we found that the NLR measured at admission was an independent predictive factor of POF in HTGP. This is because the NLR represents the inflammatory response of the patients, and organ failure occurs due to a systemic inflammatory response.

No consensus has been reached on the cut-off value to define the level of NLR in the published literature. Shelat proposed an NLR cut-off value of 3 as an indicator of prognosis in hepatocellular carcinoma patients with regards to oncologic outcomes [26]. We anticipated the NLR being higher in AP patients with a poor outcome as a result of the combination of marked neutrophilia and concomitant leucopenia seen in severe SIRS/MODS. The majority of existing studies recommend a cut-off value for NLR of ≥5 to identify poor outcomes in AP [23, 27, 28]. In this study, we found that the best cut-off value for NLR at admission was 6.56 in predicting POF in patients with HTGP, with a sensitivity of 73% and a specificity of 56%. This finding was similar to the cut-off value for predicting the severity of HTGP presented by Huang et al. [29]. Higher NLR values represent a more imbalanced inflammatory state. Compared with pancreatitis caused by other etiologies, HTGP was more likely to have severe SIRS and a poor prognosis. Previous studies suggested that HTGP was more closely related to inflammation and inflammatory imbalance [30].

The benefit of NLR is that it requires only a full blood count and is thus easier to perform, more repeatable, and does not require arterial blood gas or extended biochemistry. The greatest benefit of the NLR that differentiates it from prognostic scoring systems is the rapid confirmation of the results in an emergency examination. Furthermore, although grouped as pancreatitis, AP of various etiologies has their pathophysiology [22]. Among the etiologies, HTGP differs from others as it has a more severe clinical course and less favorable outcomes. Unfortunately, no scoring system is special for patients with HTGP. In our study, NLR, together with serum calcium, urea, and triglycerides, remained statistically significant independent predictors of POF upon presentation of the patient to the hospital. Consequently, we suggest that if larger studies are confirmatory, these variables should be considered for incorporation into established scoring systems to predict the severity of HTGP.

Evaluating the severity of AP at the initial stage of manifestation is considered the most important in the management of AP [31, 32]. Several scoring systems have been introduced over the past few decades. As one of the earliest scoring systems to predict the severity of AP, Ranson's score is shown to have comparable, if not superior, levels of accuracy as other commonly used scoring systems in stratifying severity of AP and predicting mortality [7]. While it is essential to risk stratify AP cases as early as possible, it must be noted that the clinical course of AP is dynamic and evolving. In this study, Ranson's score at 48 hours postadmission showed higher AUROC for predicting POF of HTGP than the NLR. Kiat et al. revealed that Ranson's score had higher sensitivity, negative predictive value, and AUROC for predicting the severity of AP than Glasgow score in population with predominant gallstone etiology [33]. The 48-hour time frame of Ranson's score is its inherent strength and not a weakness. It is important that there is no one perfect prognostic tool to risk stratify and predict mortality in AP patients. Clinical judgment should remain absolute in the assessment of AP, and any scoring system and predictive tool should be supplemented by clinical wisdom.

This study had several limitations. First, this was a single-center retrospective study based on medical records, which may have selection bias. Second, we did not compare NLR with other inflammatory biomarkers, such as platelet-lymphocyte ratio, CRP, and interleukin-6. Third, we did not perform dynamic observations or analyze NLR during treatment, which may reflect the progress and therapeutic response of AP [27]. Finally, due to the low prevalence of POF, the number of patients with POF was only 89. Therefore, to evaluate more accurate predictive values of NLR for POF, a prospective multicenter trial study should also be performed in addition. Despite these limitations, this study also had strengths. This study was the largest study to date evaluating the NLR as a predictor of POF in patients with HTGP. Besides, all laboratory values were obtained within one hour of initial presentation, minimizing changes in components of the total white cell count caused by hydration and medication.

## 5. Conclusions

In conclusion, our analysis indicates that the NLR may be a simple and easily accessible prognostic tool to predict POF in patients with HTGP, but still requires prospective studies to confirm in the future. NLR could be used in the future to stratify the risk of HTGP patients to offer early aggressive treatment or admission to a unit with close monitoring.

## Figures and Tables

**Table 1:** Demographic and clinical characteristics according to the occurrence of POF.

Variables	Total(*n* = 446)	Non-POF(*n* = 357)	POF(*n* = 89)	*P*
Demographics and comorbidities				
Age, median (IQR), years	38 (32-47)	39 (32-47)	36 (31-43)	0.084
Male sex, *n* (%)	325 (72.9)	255 (71.4)	70 (78.7)	0.17
BMI, median (IQR), kg/m^2^	27.0 (24.3-29.7)	26.9 (24.2-29.4)	27.7 (24.8-30.1)	0.227
Current smoker, *n* (%)	160 (35.9)	125 (35.0)	35 (39.3)	0.448
Alcohol drinker, *n* (%)	127 (28.5)	104 (29.1)	23 (25.8)	0.538
Prior acute pancreatitis, *n* (%)	213 (47.8)	179 (50.1)	34 (38.2)	0.044
Diabetes mellitus, *n* (%)	140 (31.4)	112 (31.4)	28 (31.5)	0.987
Hyperlipidemia, *n* (%)	228 (51.1)	183 (51.3)	45 (50.6)	0.906
Hypertension, *n* (%)	90 (20.2)	70 (19.6)	20 (22.5)	0.547
Time from pain onset to admission, median (IQR), hours	9.0 (5.3-20.0)	8.1 (5.0-18.0)	17.0 (7.7-28.2)	<0.001
Laboratory data at admission				
White blood cells, median (IQR), ×10^9^/L	13.1 (10.3-15.8)	12.8 (10.3-15.3)	14.9 (10.8-17.2)	0.006
Hematocrit, median (IQR), %	44.5 (40.4-47.4)	44.0 (40.0-46.8)	45.6 (41.7-49.0)	0.003
Platelet, median (IQR), ×10^9^/L	225 (177-268)	227 (177-268)	211 (178-269)	0.357
Neutrophil, median (IQR), ×10^9^/L	10.6 (8.2-13.5)	10.4 (8.1-12.9)	12.9 (8.7-14.6)	0.002
Lymphocyte, median (IQR), ×10^9^/L	1.60 (1.20-2.10)	1.60 (1.30-2.20)	1.40 (1.00-1.88)	0.006
NLR	6.56 (4.39-10.3)	6.17 (4.25-9.55)	8.51 (6.25-16.0)	<0.001
PLR	135 (102-192)	133 (101-184)	144 (103-212)	0.154
Triglyceride, median (IQR), mmol/L	20.6 (13.6-39.6)	19.8 (12.9-37.2)	26.7 (15.7-44.8)	0.005
Calcium, median (IQR), mmol/L	2.37 (2.25-2.47)	2.39 (2.30-2.48)	2.15 (1.77-2.38)	<0.001
Urea, median (IQR), mmol/L	4.24 (3.35-5.30)	4.20 (3.34-5.08)	4.80 (3.36-5.99)	0.012
Albumin, median (IQR), g/L	44.4 (41.6-47.0)	44.9 (42.8-47.2)	40.4 (35.2-44.6)	<0.001
Glucose, median (IQR), mmol/L	9.85 (7.04-15.5)	9.19 (6.92-15.2)	12.5 (8.4-16.3)	0.001
CRP, median (IQR), mg/L	14.3 (4.35-76.9)	11.6 (3.4-50.5)	65.8 (9.8-269)	<0.001
Scoring systems				
APACHE II score, median (IQR)	5 (3-8)	4 (2-7)	10 (7-12)	<0.001
Ranson's score, median (IQR)	2 (1-3)	1 (1-2)	5 (3-6)	<0.001
SOFA score, median (IQR)	1 (0-2)	1 (0-1)	2 (2-3)	<0.001
BISAP score, median (IQR)	1 (0-1)	1 (0-1)	2 (1-2)	<0.001

APACHE: acute physiology and chronic health evaluation; BISAP: bedside index of severity in acute pancreatitis; BMI: body mass index; CRP: C reactive protein; IQR: interquartile range; NLR: neutrophil-lymphocyte ratio; PLR: platelet-lymphocyte ratio; POF: persistent organ failure; SOFA: sequential organ failure assessment.

**Table 2:** Univariate and multivariate logistic regression analyses for persistent organ failure.

Variables	Univariable		Multivariable	
	OR (95% CI)	*P*	OR (95% CI)	*P*
NLR (>6.56)	3.122 (1.889-5.160)	<0.001	2.58 (1.439-4.626)	0.001
Calcium (<2.0 mmol/L)	23.903 (10.872-52.554)	<0.001	15.305 (5.550-42.208)	<0.001
Urea (>7.14 mmol/L)	9.562 (3.945-23.177)	<0.001	3.988 (1.260-12.620)	0.019
Triglyceride (>22.6 mmol/L)	1.769 (1.106-2.829)	0.017	2.561 (1.399-4.687)	0.002
Prior acute pancreatitis	0.615 (0.382-0.989)	0.045		
Time from pain onset to admission (>24 h)	2.245 (1.318-3.823)	0.003		
White blood cells (>16 × 10^9^/L)∗	2.887 (1.753-4.755)	<0.001		
Hematocrit (>44%)	1.745 (1.083-2.813)	0.022		
Albumin (<32 g/L)	8.911 (2.964-26.795)	<0.001		
CRP (>150 mg/L)	4.995 (2.924-8.534)	<0.001		
Glucose (>8.33 mmol/L)	2.346 (1.378-3.997)	0.002		

∗As white blood cells were not independent of NLR, they were excluded from the multivariate analysis. CI: confidence interval; CRP: C reactive protein; NLR: neutrophil-lymphocyte ratio; OR: odds ratio.

**Table 3:** Clinical course by NLR group.

Variables	NLR ≤ 6.56(*n* = 227)	NLR > 6.56(*n* = 219)	*P*
Atlanta classification, *n* (%)			<0.001
Mild	128 (56.4)	77 (35.2)	
Moderately severe	73 (32.2)	79 (36.1)	
Severe	26 (11.5)	63 (28.8)	
Organ support, n (%)			
Ventilation support	7 (3.1)	22 (10.0)	0.003
Continuous renal replacement therapy	10 (4.4)	30 (13.7)	0.001
Vasoactive agent	3 (1.3)	5 (2.3)	0.444
Local complication, *n* (%)			
APFC/PPC	80 (35.2)	128 (58.4)	<0.001
ANC/WON	16 (7.0)	37 (16.9)	0.001
IPN	4 (1.8)	8 (3.7)	0.217
Need for percutaneous catheter drainage, *n* (%)	7 (3.1)	12 (5.5)	0.21
Need for surgical necrosectomy, *n* (%)	0 (0)	3 (1.4)	0.077
ICU admission, *n* (%)	12 (5.3)	32 (14.6)	0.001
Hospital stay, median (IQR), days	6.18 (5.06-8.66)	7.95 (5.83-13.01)	<0.001
In-hospital mortality, *n* (%)	1 (0.4)	3 (1.4)	0.298

ANC: acute necrotic collection; APFC: acute peripancreatic fluid collection; IPN: infected pancreatic necrosis; IQR: interquartile range; NLR: neutrophil-lymphocyte ratio; PPC: pancreatic pseudocyst; WON: walled-off necrosis.

## Data Availability

The data used to support the findings of this study are available from the corresponding author upon reasonable request.

## References

[1] Jin M., Bai X., Chen X. (2019). A 16-year trend of etiology in acute pancreatitis: the increasing proportion of hypertriglyceridemia-associated acute pancreatitis and its adverse effect on prognosis. *Journal of Clinical Lipidology*.

[2] Yang A. L., McNabb-Baltar J. (2020). Hypertriglyceridemia and acute pancreatitis. *Pancreatology*.

[3] Sue L. Y., Batech M., Yadav D. (2017). Effect of serum triglycerides on clinical outcomes in acute pancreatitis: findings from a regional integrated health care system. *Pancreas*.

[4] Hines O. J., Pandol S. J. (2019). Management of severe acute pancreatitis. *BMJ*.

[5] Pavlidis T. E., Pavlidis E. T., Sakantamis A. K. (2010). Advances in prognostic factors in acute pancreatitis: a mini-review. *Hepatobiliary & Pancreatic Diseases International*.

[6] Teng T. Z. J., Tan J. K. T., Baey S. (2021). Sequential organ failure assessment score is superior to other prognostic indices in acute pancreatitis. *World Journal of Critical Care Medicine*.

[7] Ong Y., Shelat V. G. (2021). Ranson score to stratify severity in acute pancreatitis remains valid - old is gold. *Expert Review of Gastroenterology & Hepatology*.

[8] Lu Z., Zhang G., Guo F. (2020). Elevated triglycerides on admission positively correlate with the severity of hypertriglyceridaemic pancreatitis. *International Journal of Clinical Practice*.

[9] Stirling A. D., Moran N. R., Kelly M. E., Ridgway P. F., Conlon K. C. (2017). The predictive value of C-reactive protein (CRP) in acute pancreatitis - is interval change in CRP an additional indicator of severity?. *HPB: The Official Journal of the International Hepato Pancreato Biliary Association*.

[10] Zhang J., Niu J., Yang J. (2014). Interleukin-6, interleukin-8 and interleukin-10 in estimating the severity of acute pancreatitis: an updated meta-analysis. *Hepato-Gastroenterology*.

[11] Zulfic Z., Weickert C. S., Weickert T. W., Liu D., Myles N., Galletly C. (2020). Neutrophil-lymphocyte ratio - a simple, accessible measure of inflammation, morbidity and prognosis in psychiatric disorders?. *Australasian Psychiatry*.

[12] Liu Y., Zheng J., Zhang D., Jing L. (2019). Neutrophil-lymphocyte ratio and plasma lactate predict 28-day mortality in patients with sepsis. *Journal of Clinical Laboratory Analysis*.

[13] Angkananard T., Anothaisintawee T., McEvoy M., Attia J., Thakkinstian A. (2018). Neutrophil lymphocyte ratio and cardiovascular disease risk: a systematic review and meta-analysis. *BioMed Research International*.

[14] Chen Z. Q., Yu X. S., Mao L. J. (2021). Prognostic value of neutrophil-lymphocyte ratio in critically ill patients with cancer: a propensity score matching study. *Clinical & Translational Oncology*.

[15] Kong W., He Y., Bao H., Zhang W., Wang X. (2020). Diagnostic value of neutrophil-lymphocyte ratio for predicting the severity of acute pancreatitis: a meta-analysis. *Disease Markers*.

[16] Banks P. A., Bollen T. L., Dervenis C. (2013). Classification of acute pancreatitis--2012: revision of the Atlanta classification and definitions by international consensus. *Gut*.

[17] Scherer J., Singh V. P., Pitchumoni C. S., Yadav D. (2014). Issues in hypertriglyceridemic pancreatitis: an update. *Journal of Clinical Gastroenterology*.

[18] Yang Z. W., Meng X. X., Xu P. (2015). Central role of neutrophil in the pathogenesis of severe acute pancreatitis. *Journal of Cellular and Molecular Medicine*.

[19] Demols A., Le Moine O., Desalle F., Quertinmont E., van Laethem J.-L., Devière J. (2000). CD4^+^ T cells play an important role in acute experimental pancreatitis in mice. *Gastroenterology*.

[20] de Jager C. P., van Wijk P. T., Mathoera R. B., de Jongh-Leuvenink J., van der Poll T., Wever P. C. (2010). Lymphocytopenia and neutrophil-lymphocyte count ratio predict bacteremia better than conventional infection markers in an emergency care unit. *Critical Care*.

[21] Zahorec R. (2017). Neutrophil-to-lymphocyte ratio. Sixteen-year-long history since publication of our article in Bratislava Medical Journal. *Bratislava Medical Journal*.

[22] Lee P. J., Papachristou G. I. (2019). New insights into acute pancreatitis. *Nature Reviews. Gastroenterology & Hepatology*.

[23] Azab B., Jaglall N., Atallah J. P. (2011). Neutrophil-lymphocyte ratio as a predictor of adverse outcomes of acute pancreatitis. *Pancreatology*.

[24] Suppiah A., Malde D., Arab T. (2013). The prognostic value of the neutrophil-lymphocyte ratio (NLR) in acute pancreatitis: identification of an optimal NLR. *Journal of Gastrointestinal Surgery*.

[25] Wang Y., Fuentes H. E., Attar B. M., Jaiswal P., Demetria M. (2017). Evaluation of the prognostic value of neutrophil to lymphocyte ratio in patients with hypertriglyceridemia-induced acute pancreatitis. *Pancreatology*.

[26] Shelat V. G. (2020). Role of inflammatory indices in management of hepatocellular carcinoma-neutrophil to lymphocyte ratio. *Annals of Translational Medicine*.

[27] Jeon T. J., Park J. Y. (2017). Clinical significance of the neutrophil-lymphocyte ratio as an early predictive marker for adverse outcomes in patients with acute pancreatitis. *World Journal of Gastroenterology*.

[28] Cho S. K., Jung S., Lee K. J., Kim J. W. (2018). Neutrophil to lymphocyte ratio and platelet to lymphocyte ratio can predict the severity of gallstone pancreatitis. *BMC Gastroenterol*.

[29] Huang L., Chen C., Yang L., Wan R., Hu G. (2019). Neutrophil-to-lymphocyte ratio can specifically predict the severity of hypertriglyceridemia-induced acute pancreatitis compared with white blood cell. *Journal of Clinical Laboratory Analysis*.

[30] Tsuang W., Navaneethan U., Ruiz L., Palascak J. B., Gelrud A. (2009). Hypertriglyceridemic pancreatitis: presentation and management. *The American Journal of Gastroenterology*.

[31] Rawla P., Sunkara T., Thandra K. C., Gaduputi V. (2018). Hypertriglyceridemia-induced pancreatitis: updated review of current treatment and preventive strategies. *Clinical Journal of Gastroenterology*.

[32] Lu Z., Li M., Guo F. (2020). Timely reduction of triglyceride levels is associated with decreased persistent organ failure in hypertriglyceridemic pancreatitis. *Pancreas*.

[33] Kiat T. T. J., Gunasekaran S. K., Junnarkar S. P., Low J. K., Woon W., Shelat V. G. (2018). Are traditional scoring systems for severity stratification of acute pancreatitis sufficient?. *Annals of Hepato-Biliary-Pancreatic Surgery*.

